# Aflibercept in branch retinal vein occlusion as second line therapy: clinical outcome 12 months after changing treatment from bevacizumab/ranibizumab—a pilot study

**DOI:** 10.1186/s40942-016-0045-8

**Published:** 2016-08-23

**Authors:** Magdalena A. Wirth, Matthias D. Becker, Nicole Graf, Stephan Michels

**Affiliations:** 1 Department of Ophthalmology, City Hospital Triemli Zurich, Zurich, Switzerland; 2 Department of Ophthalmology, University of Heidelberg, Heidelberg, Germany; 3 Graf Biostatistics, Winterthur, Switzerland; 4 Department of Ophthalmology, University of Zurich, Zurich, Switzerland

**Keywords:** Anti-VEGF, Aflibercept, Branch retinal vein occlusion, Macular edema

## Abstract

**Purpose:**

To evaluate the effect of aflibercept (as second line therapy) on the clinical outcome in patients with chronic macular edema secondary to branch retinal vein occlusion (BRVO) insufficiently responding to prior treatment with bevacizumab and/or ranibizumab.

**Methods:**

Ten eyes of ten patients (n = 10) with chronic macular edema secondary to BRVO were included in a retrospective analysis. These patients received aflibercept after an insufficient response to treatment with ranibizumab and/- or bevacizumab. All intravitreal injections were administered according to a “treat and extend” regimen. Insufficient response was defined as the necessity of injection intervals of 6 weeks or less. The primary outcome of the study was the change in mean injection interval from baseline (prior switching to aflibercept) to month 12 after conversion to aflibercept. Secondary outcomes included the change in best corrected visual acuity (BCVA), central retinal thickness (CRT), central retinal volume (CRV) and intraocular pressure (IOP).

**Results:**

All patients completed 12 months follow-up. In total, patients received a mean of 15.5 injections of ranibizumab and/or bevacizumab over a mean period of 23.1 months prior to switching to aflibercept. The primary endpoint indicated a significant increase in the injection interval from 5.0 weeks at baseline to 8.3 weeks at month 12 (p = 0.002). Secondary outcomes showed favorable results. Mean BCVA increased from 72.7 letters at baseline to 77.9 letters at month 12 after treatment initiation with aflibercept (+5.2 letters, p = 0.375). Correspondingly, CRT values decreased by 61.7 µm (p = 0.344) and the mean CRV (6 mm diameter) by 0.86 mm^3^ (p = 0.021) from baseline to 1 year after treatment initiation with aflibercept. During the treatment period with aflibercept no significant changes in intraocular pressure were registered (p = 0.238).

**Conclusions:**

Changing treatment to aflibercept in patients with chronic macular edema secondary to BRVO showed a statistically significant extension of the retreatment interval as well as beneficial anatomic changes in our study group. Our data do not allow a definite conclusion since the study was not controlled.

## Background

Among retinal vascular pathologies branch retinal vein occlusion (BRVO) was reported to be the second most common entity after diabetic retinopathy, with a cumulative 15-year incidence of 1.8 % [1]. BRVO mostly occurs at arterio-venous crossing sites, explaining the contribution of arteriosclerosis in adjacent arteries [2]. It is associated with cardiovascular risk factors and pathogenically follows the principle of Virchow’s triad (stasis, hypercoagulability and endothelial damage) [3]. BRVO is frequently associated with macular edema (ME) as the endothelial damage results in an inflammatory response of affected vessels with subsequent up-regulation of inflammatory mediators such as TNF-alpha, prostaglandins, leukotriens, integrins and vascular endothelial growth factor (VEGF) [4, 5]. Studies showed that VEGF plays a major role in the evolution and persistence of secondary ME [6]. Earlier treatment of ME was reported to be associated with better results in terms of long-term visual acuity [7].

For many years, the gold standard treatment for macular edema in BRVO was grid laser photocoagulation [8]. With the advent of anti-VEGF compounds first-line treatment strategies of ME in BRVO are intravitreal anti-VEGF agents [ranibizumab (Lucentis ©, Novartis), bevacizumab (Avastin ©, Genentech) and aflibercept (Eylea ©, Regeneron Pharmaceuticals Inc.)]. In comparison to grid laser photocoagulation, the use of aflibercept led to a significant visual benefit and reduction in central retinal thickness (CRT) in eyes with macular edema secondary to BRVO as reported in the VIBRANT study [9].

Aflibercept, the most recently developed anti-VEGF agent, is a 115-kDa soluble receptor fusion protein consisting of the second domain of VEGF receptor 1 and the third domain of VEGF receptor 2 fused to the Fc domain of immunoglobulin G1. In contrast to other anti-VEGF agents, aflibercept additionally binds PGF (placental derived growth factor) and has a considerably higher binding affinity for VEGF [10, 11]. Furthermore a longer duration of action was reported, which contributed to the recent approval for macular edema in CRVO [12]. Experience with aflibercept in patients with CRVO and ME insufficiently responding to prior anti-VEGF showed favorable outcomes [13, 14].

The current study was designed to evaluate the change of injection interval and clinical outcomes in patients who have been treated with aflibercept for at least 12 months as a consequence of an insufficient response to ranibizumab and/or bevacizumab.

## Methods

This retrospective clinical study was conducted at the department of ophthalmology, City hospital Triemli Zurich and was approved by the local ethics committee (Ethics Commission of the Canton Zurich, KEK-ZH-Nr. 2014-0601). Its conduction adhered to the tenets of the declaration of Helsinki. Included patients (n = 10) gave their written informed consent on retrospective data evaluation and its publication. Data were retrieved from an internal database, containing clinical information of all patients treated with anti-VEGF agents during the past 3 years (2012–2015). In addition medical charts and optical coherence tomography scans (OCT) of included patients were reviewed.

Patients of Caucasian descent in whom therapy was changed to off-label aflibercept as an individual case decision, after an insufficient response to treatment with bevacizumab or ranibizumab, were included in this case series. Insufficient response was defined as the necessity of injection intervals of 6 weeks or less, elicited by the persistence or any increase of intraretinal fluid as imaged by spectral-domain optical coherence tomography (Heidelberg Spectralis **©** System). Evidence of increasing or persistent fluid in any of the 19 standard scans (512 A—scans, 20° × 15°) was considered as disease-related activity.

All intravitreal injections were administered according to a “treat and extend” regimen [15]. This protocol is characterized by the adjustment of individual injection intervals according to therapeutic response. The main objective of this protocol is to reduce the treatment burden for the patient, considering risks and inconveniences each injection carries, as well as economic aspects. At each visit OCT scans were evaluated for alterations of intraretinal and subretinal fluid. The absence of any activity sign (increasing and/or persistent fluid) resulted in a prolongation of the current injection interval by 2 weeks and vice versa.

The primary outcome was the change in the mean injection interval comparing baseline (prior to treatment with aflibercept) to month 12 after switching to aflibercept. Secondary outcomes included: best-corrected visual acuity (BCVA), central retinal thickness (CRT), central retinal volume (CRV) (6 mm diameter, 512 A—scans, 20° × 15°) and intraocular pressure (IOP, as measured by air-puff tonometry). For statistical purposes, visual acuity measures (registered as Snellen acuity fractions) were converted into ETDRS scores (Early Treatment Diabetic Retinopathy Study) as described by Gregori et al. [16]. Information on CRT and CRV was retrieved from spectral- domain optical coherence tomography (SD-OCT) scans (Heidelberg Spectralis © System, Heidelberg Engineering). OCT images at month 6 and 12 were taken using the SLO-based (scanning laser ophthalmoscope) eye tracking system (AutoRescan), ensuring the correct location for follow-up.

Statistical analyses were performed using SPSS^**®**^ Version 20 and Microsoft Office Excel for Windows, Version 2007. Binomial and exact Wilcoxon signed rank tests were employed for asymmetrical (visual acuity, central retinal thickness and volume, injection interval) and symmetrical (IOP) distributions of differences, respectively.

## Results

Ten eyes (n = 10) of ten patients with macular edema secondary to BRVO were included in the current retrospective analysis. These patients received aflibercept after an initial insufficient response (criteria see above) to treatment with ranibizumab and/or bevacizumab.

The study group consisted of 8 male and 2 female patients. Mean age at baseline amounted to 73.9 ± 5.2 years.

Eight patients were pretreated with ranibizumab, 1 patient was pretreated with bevacizumab intravitreal injections and 1 patient was pretreated with both anti-VEGF agents. At baseline (pre-aflibercept), patients had received a mean of 15.5 (SD ± 5.02) injections during a mean period of 23.1 (SD ± 13.8) months (range 10–53 months). During the 10–12 months prior switching treatment to aflibercept, included patients received a mean of 10.0 injections.

**Fig. 1:**
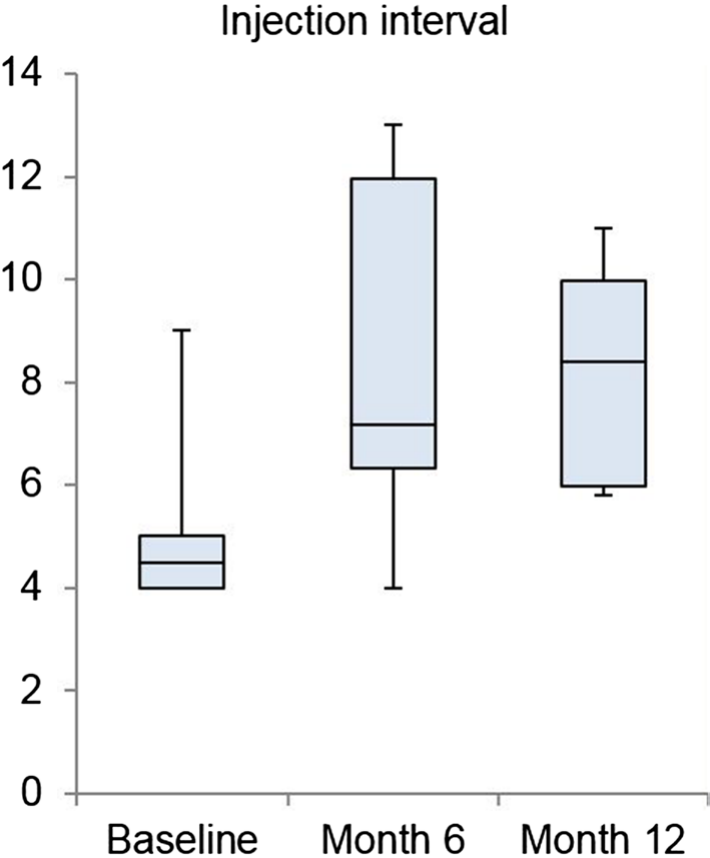
Injection interval (median, SD): change from baseline to month 12 (p = 0.002)

The mean injection interval—as primary outcome—increased from 5.0 ± 1.6 weeks at baseline (prior therapy change) to 8.5 ± 3.3 weeks at month 6 and 8.3 ± 2.1 weeks at month 12 (p = 0.002) (Fig. 1). This resulted in a mean prolongation of 3.3 ± 1.8 weeks throughout the observation period.

**Fig. 2:**
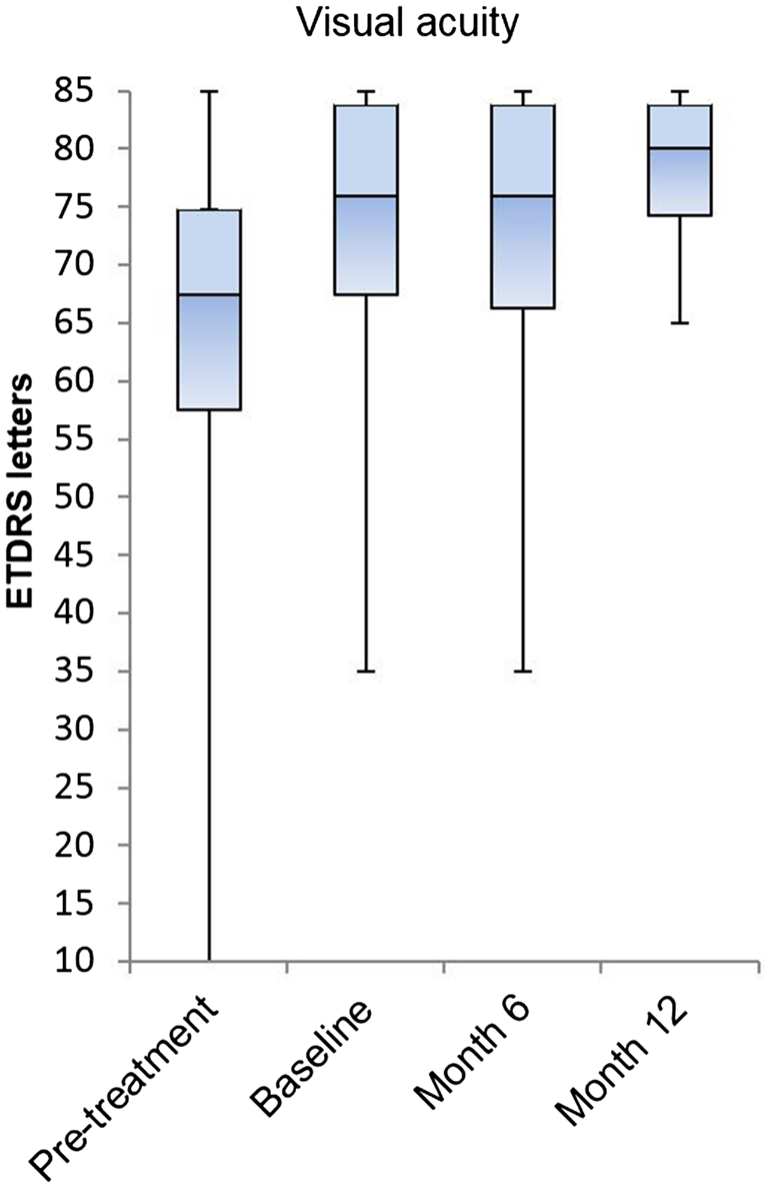
Visual acuity (median, SD): change from baseline to month 12 (p = 0.375)

As functional and secondary outcome, BCVA fluctuated from 72.7 ± 15.2 letters at baseline to 72.2 ± 15.2 letters at month 6 and 77.9 ± 6.8 letters at month 12 (Fig. 2). The gain of 5.2 (SD ± 11.0) letters after 12 months did not reach the level of statistical significance (p = 0.375). From initial diagnosis to baseline (therapy switch to aflibercept) a mean gain of 10.3 ± 11.0 letters was measured.

**Fig. 3:**
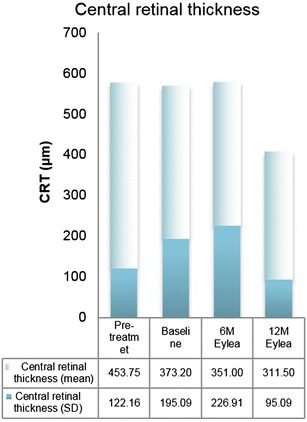
Central retinal thickness (mean, SD): change from baseline to month 12 (p = 0.344)

**Fig. 4:**
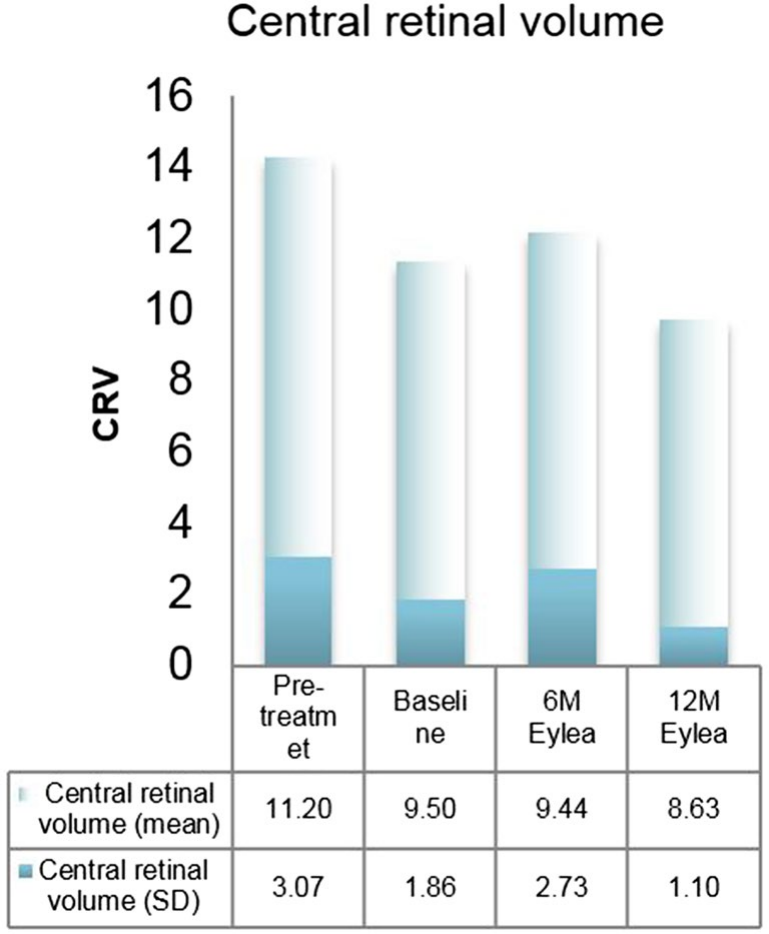
Central retinal volume (mean, SD): change from baseline to month 12 (p = 0.021)

Secondary anatomical outcomes, such as the mean CRT and mean CRV likewise indicated an improvement. Pretreatment CRT amounted to 453.8 ± 122.2 µm. CRT decreased from 373.2 ± 195.1 µm at baseline to 351.0 ± 226.9 µm at month 6 and 311.5 ± 95.1 µm at month 12. The mean reduction of 61.7 µm ± 192.1 did not reach the level of statistical significance (p = 0.344) (Fig. 3). Analogously, CRV was reduced from 9.5 ± 1.9 mm^3^ at baseline to 9.4 ± 2.7 mm^3^ at month 6 and 8.6 ± 1.1 mm^3^ at month 12. This resulted in a mean reduction of 0.9 ± 2.4 mm^3^ within the treatment period of 12 months (p = 0.021). Pretreatment values amounted to 11.2 ± 3.1 mm^3^ (Fig. 4).

Intraocular pressure values were not significantly affected by aflibercept intravitreal injections during the study period (p = 0.238).

## Discussion

Our data indicate that by switching anti-VEGF therapy to aflibercept in eyes with chronic, recurrent ME due to BRVO a significant extension of the injection interval can be obtained. Our data do not allow a definite conclusion since the study was not controlled and included only a small number of patients. However, it appears rather unlikely that a chronic recurrent ME in BRVO shows on average almost half the need for treatment within 6 months (extension of treatment intervals by a mean of 3.5 weeks). Nevertheless, the substantive range of injection intervals (±3.3 weeks) at month 12 must be considered concerning this matter. The further follow-up (month 6–12) showed no further relevant change in the treatment interval, indicating that rather the change in therapy than a continuous regression of disease activity is the origin of extended treatment intervals. A spontaneous regression in the natural course of macular edema secondary to BRVO as described by Rogers et al. [17] appears rather unlikely since patients with recurrent chronic ME were selected.

The rationale for this finding is likely related to different properties of aflibercept (higher VEGF binding affinity, additional PGF binding) compared to ranibizumab and bevacizumab [10, 11]. Recently presented data demonstrated the longest intravitreal retention time for aflibercept in comparison to bevacizumab and ranibizumab [18]. Analysis of intraocular VEGF levels in humans following intravitreal aflibercept administration indicated that VEGF was suppressed below the lower limit of quantification for 10 weeks on average [19].

Functional and anatomic outcomes (CRT, CRV, BCVA) showed some improvement during the whole study period after therapy change to aflibercept (baseline to month 12). However, only a minority of parameters showed statistically significant changes. This has to be seen in the context that all eyes had been extensively treated prior to change of therapy and that they had a significant increase in their visual acuity (+10.3 letters) from prior to any therapy to baseline. Therefore, the additional potential functional and anatomic gain already was limited.

Since only two patients were pretreated with bevacizumab, comparison of outcomes between the two types of anti-VEGF pretreatments (ranibizumab/bevacizumab) was not judged as expedient in our analysis.

Due to lack of data for aflibercept in BRVO, no comparison with existing studies can be made. Pfau et al. indicate superior results of aflibercept in CRVO as compared to prior ranibizumab/bevacizumab treatment [13]. Since edema secondary to BRVO in the majority of cases is less pronounced than in CRVO, a less measurable improvement following treatment may be a consequence.

## Conclusions

In conclusion, our retrospective analysis showed a statistically significant and clinically relevant prolongation of injection intervals within 12 months following a change of anti-VEGF therapy in patients with chronic recurrent ME secondary to BRVO. However, improvements regarding BCVA and CRT were limited. Larger prospective, clinical trials are required not only to confirm less need for treatment with aflibercept, but also to clearly demonstrate a functional and anatomic benefit by changing anti-VEGF agents.

### Limitations

The small number of patients, the lack of a control group and the retrospective study design not only inhere the risk of type 1 and type 2 errors, but also of selection and performance bias. Moreover, interobserver variability must be mentioned as a potential source of bias.

## Abbreviations

BRVO: branch retinal vein occlusion
ME: macular edema
PED: pigment epithelial detachment
VEGF: vascular endothelial growth factor
PGF: placental derived growth factor
CRT: central retinal thickness
CRV: central retinal volume
BCVA: best corrected visual acuity
IOP: intraocular pressure
SD-OCT: spectral domain optical coherence tomography
SLO: scanning laser ophthalmoscope
SD: standard deviation
ETDRS: early treatment diabetic retinopathy study

## References

[1] Klein R, Moss SE, Meuer SM, Klein BE (2008). The 15-year cumulative incidence of retinal vein occlusion: the Beaver Dam Eye Study. Arch Ophthalmol..

[2] Haymore JG, Mejico LJ (2009). Retinal vascular occlusion syndromes. Int Ophthalmol Clin..

[3] Encke A (1977). Pathophysiology of venous thrombosis (author’s transl). Langenbecks Arch Chir.

[4] Rehak M, Wiedemann P (2010). Retinal vein thrombosis: pathogenesis and management. J Thromb Haemost.

[5] Deobhakta A, Chang LK (2013). Inflammation in retinal vein occlusion. Int J Inflam.

[6] Campochiaro PA (2013). Vascular endothelial growth factor promotes progressive retinal nonperfusion in patients with retinal vein occlusion. Ophthalmology.

[7] Thach AB (2014). Time to clinically significant visual acuity gains after ranibizumab treatment for retinal vein occlusion: BRAVO and CRUISE trials. Ophthalmology.

[8] Bressler NM, Schachat AP (2010). Management of macular edema from retinal vein occlusions: you can never have too many choices. Ophthalmology.

[9] Campochiaro PA (2015). Intravitreal aflibercept for macular edema following branch retinal vein occlusion: the 24-week results of the VIBRANT Study. Ophthalmology.

[10] Papadopoulos N (2012). Binding and neutralization of vascular endothelial growth factor (VEGF) and related ligands by VEGF Trap, ranibizumab and bevacizumab. Angiogenesis.

[11] Holash J (2002). VEGF-Trap: a VEGF blocker with potent antitumor effects. Proc Natl Acad Sci U S A.

[12] Evoy KE, Abel SR (2013). Aflibercept: newly approved for the treatment of macular edema following central retinal vein occlusion. Ann Pharmacother.

[13] Pfau M (2015). Clinical outcome after switching therapy from ranibizumab and/or bevacizumab to aflibercept in central retinal vein occlusion. Ophthalmic Res.

[14] Papakostas TD (2016). Intravitreal aflibercept for macular oedema secondary to central retinal vein occlusion in patients with prior treatment with bevacizumab or ranibizumab. Eye (Lond).

[15] Freund KB (2015). Treat-and-extend regimens with anti-VEGF agents in retinal diseases: a literature review and consensus recommendations. Retina.

[16] Gregori NZ, Feuer W, Rosenfeld PJ (2010). Novel method for analyzing snellen visual acuity measurements. Retina.

[17] Rogers SL (2010). Natural history of branch retinal vein occlusion: an evidence-based systematic review. Ophthalmology.

[18] Christoforidis JB (2012). Pharmacokinetic properties of intravitreal I-124-aflibercept in a rabbit model using PET/CT. Curr Eye Res.

[19] Fauser S, Schwabecker V, Muether PS (2014). Suppression of intraocular vascular endothelial growth factor during aflibercept treatment of age-related macular degeneration. Am J Ophthalmol.

